# Improvement of Electroacupuncture on APP/PS1 Transgenic Mice in Spatial Learning and Memory Probably due to Expression of A*β* and LRP1 in Hippocampus

**DOI:** 10.1155/2016/7603975

**Published:** 2016-10-18

**Authors:** Xin Wang, Yanhuan Miao, Jiawula Abulizi, Fu Li, Yuping Mo, Weiguo Xue, Zhigang Li

**Affiliations:** ^1^School of Acupuncture, Moxibustion and Tuina, Beijing University of Chinese Medicine, No. 11 Bei San Huan Dong Lu, Chaoyang District, Beijing 100029, China; ^2^Community Health Service Administration Center of Dongcheng District, No. 192-1 Chaoyangmen Internal Street, Dongcheng District, Beijing 100053, China

## Abstract

*Objectives*. To explore the alterations of *β*-amyloid (A*β*) and low density lipoprotein receptor-related protein-1 (LRP1) in APP/PS1 mice after electroacupuncture (EA) treatment and further to explore the mechanism.* Methods*. Forty 6-month-old APP/PS1 mice were randomly divided into a model group and an EA group, with twenty wild-type mice used as a normal control group. Mice in the EA group were treated with EA at GV 20 (*băi huì*) and bilateral KI 1 (*yŏng quán*) acupoints for 6 weeks. The Morris water maze was applied to assess the spatial memory in behavior. Immunohistochemistry (IHC), ELISA, Western blotting, and so forth were used to observe the expression of LRP1 and A*β*.* Results*. The Morris water maze test showed that, compared with the normal control group, the model group's learning and memory capabilities were significantly decreased (*P* < 0.05; *P* < 0.01). The EA group was reversed (*P* < 0.05; *P* < 0.01). The hippocampal expression of A*β* in the EA group was significantly decreased compared to the model group (*P* < 0.01). The expression of LRP1 in the model group was significantly lower than that in the normal control group (*P* < 0.01); the expression in the EA group was significantly higher than that in the model group (*P* < 0.01).* Conclusions*. EA therapy can improve the learning and memory capabilities of APP/PS1 mice. The underlying mechanism may lie in the upregulation of an A*β* transport receptor and LRP1.

## 1. Introduction

Alzheimer's disease (AD), the most common cause of dementia in the elderly population, is a kind of neurodegenerative diseases mainly indicated by progressive cognition and memory impairment [1, 2].

In recent decades, the pathological mechanisms of AD have been widely studied [3, 4]. The accumulation of amyloid *β*-peptide (A*β*) plays the most important role in the pathogenesis of AD [5, 6]. A wealth of evidence has indicated that A*β*_1–42_ deposits participate in the process of neuronal loss which then leads to the occurrence of dementia in AD patients [7]. The high level of A*β* in sporadic AD lies in the imbalance of A*β* production and clearance, especially disordered A*β* clearance [8]. Recent research has focused on A*β* clearance pathways through the cranial microvascular saturable efflux system, namely, transport across the blood-brain barrier (BBB), which has both a fast transport speed and large transport volume. Therefore, the relationship between brain microvessels and the clearance of A*β* is also crucial [9, 10].

A leading hypothesis supports the fact that the main clearing pathway of A*β* in the brain is the transportation of A*β* through the BBB into peripheral blood which has a strong clearing ability to it [5, 11, 12]. In addition, the BBB model cultured in vitro by the brain microvascular endothelial cell line has also been used to detect whether A*β* can be transported through the BBB, and the finding was affirmative [13, 14]. A*β* is a polar, soluble macromolecular substance [15], and it cannot be freely exchanged between the brain and peripheral blood via free diffusion. Therefore, if A*β* transportation across BBB exists, there must be A*β* specific transporters in the BBB.

Low density lipoprotein receptor-related protein-1 (LRP1) is known to function as a BBB clearance (or efflux) transporter for A*β*. Efflux of A*β* is initiated when it binds directly to LRP1 at the abluminal membrane of the brain endothelial cell [16]. Yamada et al. [17] proved that the brain microvascular endothelial cell uptaking A*β* mainly relies on LRP1 under the BBB-specific cellular context. Bell et al. [18] found in animal experiments that the isotope-labeled A*β* injected into the caudate nucleus of mice would be cleared out from the brain rapidly, and the labeled A*β* was found in the plasma. The clearance of A*β* could be inhibited by LRP1 specific antibodies. Further studies [16, 19, 20] suggest that, in pathological conditions, the abnormality of A*β* levels in the brain might be associated with the altered expression of LRP1 in cranial microvessels.

One survey reported that 55% of patients with AD had tried at least one form of complementary medicine with the hopes that these therapies could improve their overall quality of life and delay further decline in cognitive functioning. Clinical research has shown that acupuncture can improve the mental and behavioral conditions of AD patients, as well as the cognitive function [21–23]. Electroacupuncture (EA) is a simple and effective modern acupuncture method used in the treatment of many diseases. A previous study has reported that EA at GV 20 (*băi huì*) shows a significant protective effect on neuronal damage and impairment of learning and memory [24]. More evidences have proven that acupuncture has a therapeutic effect on AD [25–27]. However, the mechanism is still unclear and more exploration is needed.

In this study, 6-month-old APP/PS1 transgenic mice were selected as the animal model of AD. EA at GV 20 (*băi huì*) and KI 1 (*yŏng quán*) was given to the mice. The effects on their behavior and the expression of A*β*_1–42_ and LRP1 levels in the hippocampus were observed and analyzed so as to explore the treatment mechanism of EA on early intervention of AD.

## 2. Materials

### 2.1. Animal Model and Grouping

#### 2.1.1. Animals

6-month-old APPswe/PS1dE9 double transgenic male mice were used as the animal model of AD, and wild-type mice with the same age and sex were used as the normal control group. Animals were purchased from Model Animal Research Center of Nanjing University (animal lot: SCXK (Ning) 2010-000), weighing 34.2 ± 3.98 g. All experimental procedures comply with the guidelines of the “Principles of Laboratory Animal Care” formulated by the National Institute of Health and the legislation of the People's Republic of China for the use and care of laboratory animals. The experimental protocols were approved by the Animal Experimentation Ethics Committee of Beijing University of Chinese Medicine. Efforts were made to minimize the number of animal uses and the suffering of the experimental animals.

#### 2.1.2. Animal Grouping and Intervention

40 APP/PS1 transgenic mice were randomly divided into two groups, a model group (M) (*n* = 20) and an electroacupuncture group (EA) (*n* = 20), while 20 wild-type mice were selected as the normal control group (C). All mice were raised with a regular diet in single cages in the barrier system of the Animal Center of Beijing University of Chinese Medicine.

Regarding EA group, EA on GV 20 (*băi huì*) and bilateral KI 1 (*yŏng quán*) was given to the mice, with transversely puncturing at a depth of 2-3 mm by disposable sterile acupuncture needles (0.25 mm × 13 mm) (Beijing Zhongyan Taihe Medicine Company, Ltd.). The anode and cathode of EA were, respectively, connected to the left and right KI 1 (*yŏng quán*), with a dilatational wave at a frequency of 2/15 Hz, 1 mA, and an intensity that the needle tremors, while animals keep quiet by Han's acupoint nerve stimulator (Beijing Huawei Industrial Development Company, Han's LH202H type). GV 20 (*băi huì*) is located at the intersection of the sagittal midline and the line linking two rat ears [26]. KI 1 (*yŏng quán*) is located on the sole of the foot, at the indentation near the front part, between the second and third metatarsal bones, 1/3 of the distance from the webs of the toes to the heel [28]. The EA treatment was performed with mice restrained in mouse bags, 15 mins per time, once every other day for 6 weeks. Mice in the normal control group and the model group were just restrained in the same mouse bags for 15 min, once every other day, totally for six weeks [29].

## 3. Methods

### 3.1. Learning and Memory Behavioral Testing

Morris water maze testing was conducted in a round pool to test the behavior of learning and memory [30]. Water maze training was given on the 2nd–6th days after EA intervention for 6 weeks, with water temperature at 22  ±  2°C. One day before the experiment, mice in each group were placed on the platform for 10 s to adapt to the environment, and each mouse was offered a period of free swimming in the maze for 1 min with the fourth quadrant as the entry point. On the first to fourth days of the experiment, mice in each group were given a place navigation test. Mice were firstly placed on the platform to adapt to the environment for 10 s and then were sequentially put into water facing the wall away from the four quadrants in sequence. The time recording was stopped after 5 s of the mouse on the platform, and the maximum swim time was set at 60 s. On the fifth day, the spatial probe test was performed. The platform was removed, and mice were sequentially placed directly into the water from the four quadrants. A computer connected to an image analyzer (BS-124S Morris water maze video analysis system: provided by the Pharmacology Laboratory of Dongzhimen Hospital affiliated to Beijing University of Chinese Medicine, Shanghai Mobile Information Technology Co., Ltd.) monitored the swim pattern.

### 3.2. Collection of Brain Tissues and Detection of Related Indexes

#### 3.2.1. A*β*_1–42_ Immunohistochemistry

*Sample Preparation*. The brains of 2 mice in each group were fixed in paraformaldehyde after cardiac perfusion and then trimmed, dehydrated with ethanol, made transparent with xylene, embedded in paraffin, and sectioned on a coronal plane.

*Aβ*_*1–42*_ *Immunohistochemical ABC Method*. Sections were firstly dewaxed and hydrated and were put into 0.01 mol/L citrate buffer for antigen thermal remediation for 10 min and 3% methanol hydrogen peroxide at room temperature for 10 min. Then the sections were blocked in 5% normal goat serum at 37°C for 30 min and incubated with primary antibody diluent (USA, Abcam, ab10148, 1 : 100) for one night at 4°C. After incubation within the primary antibody diluent, the sections were rinsed with phosphate-buffered saline (PBS) and then incubated with secondary antibody diluent (Boster Biological Engineering, goat anti-rabbit HRP-IgG, 1 : 1000) for 90 minutes at 37°C. The sections were rinsed with PBS before incubation with AB complex for 90 minutes at 37°C and were placed into diaminobenzidine (DAB) solution for 10 minutes after being rinsed another time with PBS. After being redyed with hematoxylin, they were dehydrated and mounted after transparence. The sections were observed under the microscope (BX53, Olympus Corporation, Japan).

#### 3.2.2. Laser Confocal Imaging of Frozen Section Observation with Laser Scanning Confocal Microscope

*Sample Preparation*. With 3 mice in each group, the analytes were fixed in paraformaldehyde after cardiac perfusion. 30% sucrose was then added after 24 hours, and brain tissues were frozen sectioned with OTC embedded when sunk into the bottom of the bottle.

Proteinase K was added at 37°C after the brain tissues were washed three times with PBS with 5 mins each. 30 min later, LRP1 (USA, Abcam, ab28320, 1 : 70) and A*β*_1–42_ (USA, Abcam, ab10148, 1 : 100) were added equivalently after washing for three times with PBS as above. With incubation overnight at 4°C, the second antibodies (FITC (USA, Abcam, ab6785, 1 : 250) and Alexa Fluor® 647 (USA, Abcam, ab150079, 1 : 150)) were added after washing with the same method. With incubation for 1.5 hours at indoor temperature, DAPI (Beijing Zhongshan Golden Bridge, ZLI-9557) was added to fix the sections after washing as before. A laser scanning confocal microscope (FV1000, Olympus Corporation, Japan) was used for sections observation.

### 3.3. Collection and Preservation of Brain Tissues

After the water maze test, mice in each group were anesthetized with 0.3% sodium pentobarbital (30 mg/kg). The 15 hippocampi were taken with craniotomy and prepared for ELISA and Western blotting samples in time on the left and right sides, respectively, and then preserved in a refrigerator at 80°C.

### 3.4. Preparation of Samples 

#### 3.4.1. For A*β* ELISA

The 6 right hippocampi were weighed in each group and homogenized with 8 times the volume of mixed liquor of 5 M guanidine hydrochloride, 50 mM Tris hydrochloric acid (pH 8.0), and 1 mM PMSF on ice. Then the hippocampi were centrifuged with 16000 r/min, at 4°C, for 20 min, and the supernatant was obtained. The diluted samples were prepared by separately mixing the supernatant with a standard diluent of 3200 times the volume (KHB3441, Invitrogen, USA) and 800 times the volume (KHB3441, Invitrogen, USA) with the hippocampus of the model and EA groups. The samples in the normal control group were not diluted.

#### 3.4.2. For LRP1 Western Blotting

The 6 left hippocampi were added to the RIPA lysate solution containing 1 mM PMSF with the weight ratio of 1 : 100 in each group, and then the total protein was extracted from tissues after homogenizing. Then the hippocampi were centrifuged at 2000 r/min, at 4°C, for 10 minutes, after extraction, and the volume of the supernatant was calculated. The samples were then prepared for Western blotting.

#### 3.4.3. For LRP1 ELISA

The remaining 9 hippocampi were weighed in each group and homogenized with a dilution of 1 : 8 in PBS. The supernatant was extracted with the same extracting method of 6 right hippocampi. The diluted samples to be tested were prepared by adding the supernatant with a standard dilution of 1 : 5 (DRE20100, RD, USA) in all groups.

### 3.5. Double Antibody Sandwich Method for ELISA

The ELISA kit was used to, respectively, detect samples. First, 50 *μ*L of antibody operating solution was added into each plate at room temperature for 120 minutes. Then 100 *μ*L horseradish peroxidase labeled secondary antibody operating solution was added to each plate at room temperature and dark reaction for 30 minutes. Complete plate washing was performed after four attempts. A 100 *μ*L chromogenic substrate operating solution was added to each plate at room temperature and dark reaction for 30 minutes. Plate washing was performed after four completed attempts. Then 100 *μ*L stop solution was added and mixed into each plate. The absorbance was detected at the 450 nm place with a microplate reader within 30 minutes. The standard protein line was drawn with Excel. The sample concentration was converted according to the sample readings, and A*β*_1–42_, A*β*_1–40_, and LRP1 concentrations were calculated according to the sample diluted concentration.

### 3.6. Western Blotting

SDS-PAGE electrophoresis was performed with a 10% separating gel and a 5% stacking gel and transferred to a 0.45 *μ*m PVDF membrane. Membrane blocking was performed using 5% nonfat milk in Tris buffered saline supplemented with 0.1% Tween 20 (TBST). The first antibody (USA, Abcam, ab92544, 1 : 20000; ab8227, 1 : 2000) was added prior to incubation for one night at 4°C. The secondary antibody (USA, Abcam, ab6721, 1 : 2000) was added before shaking and incubating at room temperature for 1.5 h. HRP-ECL luminous liquid was added and the X-ray film exposure was completed in a dark room following the developing and fixing. After calibrating the markers, analysis and scanning were performed, and the relative expression of LRP1 (LRP1/*β*-actin gray value) was compared in each group.

## 4. Statistical Analysis

SPSS 17.0 software was used to conduct the statistical analysis. All data were presented as means ± standard deviation ($\overset{-}{x}\pm s$). Variance analysis of multigroup repeated measurement design date was adopted for the data of the Morris water maze behavioral escape latency. One-way ANOVA was used after the test of normal distribution and homogeneity of variance, and LSD method was used for pairwise comparisons for the ELISA detection and Western blotting. If there was a nonnormal distribution or heterogeneity of variance for the data, a nonparametric test would be used. Statistical significance was set to *P* < 0.05, while highly statistical significance was set to *P* < 0.01.

## 5. Results

### 5.1. Effect of EA on Spatial Learning and Memory

**Figure 1:**
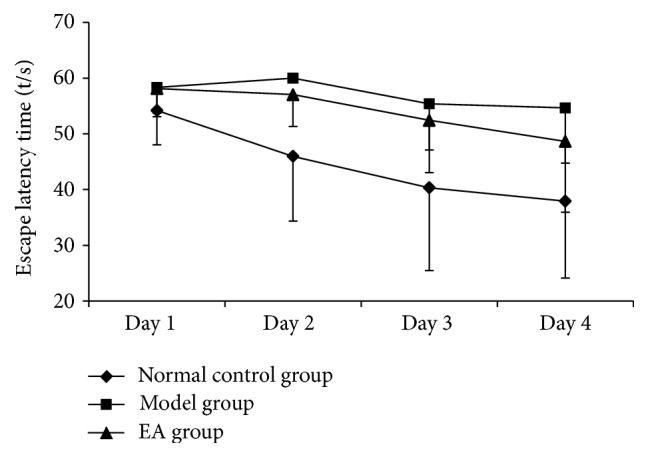
Comparison of escape latency time in each group in Morris water maze place navigation test.

Statistical results showed that, according to variance analysis of repeated measurement and effect between groups, the escape latency time in each group was significantly decreased with the increase of training days, and there were significant differences among groups (*P* < 0.05). There was a significant difference for the training time (day) (*P* < 0.05), but there was no significant difference for the interaction between the training time and groups (day × group) (*P* > 0.05). Pairwise comparison results showed that the escape latency time in the model group was significantly longer than that in the control and EA groups (see Table 1 and Figure 1).

One-way ANOVA was used to determine the number of platform crossovers and the swimming distance in the platform quadrant for the spatial probe test for the three groups, and the differences were statistically significant (*P* < 0.05). The number of platform crossovers and the swimming distance in the platform quadrant of the model group were significantly lower than those in the normal control group (*P* < 0.01). Compared with the model group, the number of platform crossovers of the EA group was significantly higher (*P* < 0.05), and the swimming distance in the platform quadrant in the EA group was significantly longer (*P* < 0.01) (see Table 2).

**Figure 2:**
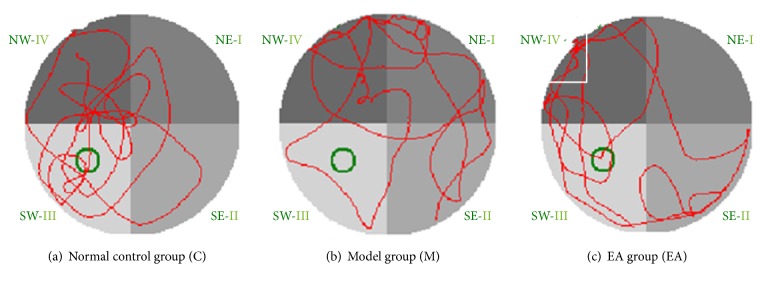
Swimming trajectories with fourth quadrant as water entry point in each group in Morris water maze spatial probe test: (a) normal control group: the swimming trajectory was mostly concentrated in the original target platform quadrant; (b) model group: the swimming trajectory was random. (c) EA group: the swimming trajectories were concentrated in the original target quadrant or adjacent quadrants.

The swimming trajectories of mice in the normal control group were mostly concentrated in the original target platform quadrant, and the subjects tended to search mainly. However, the type of searching in the model group was random. Compared to the model group, the swimming trajectories of mice in the EA group were more concentrated in the original target quadrant or adjacent quadrants, and the searching trend had a linear and trending appearance (see Figure 2).

### 5.2. Effect of EA on A*β* and LRP1

#### 5.2.1. Immunohistochemistry Results of A*β*_1–42_ in Hippocampus

**Figure 3:**
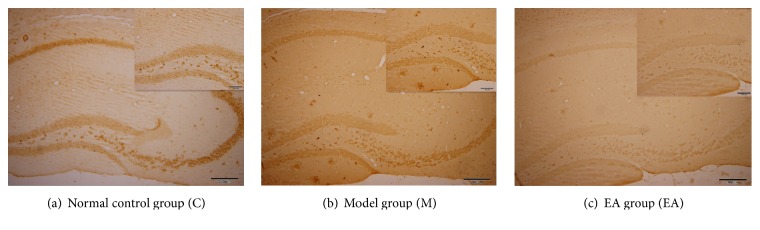
Immunohistochemistry of A*β*_1–42_: complete view (original magnification ×100, 1 : 200); sectional view (original magnification ×200, 1 : 100); (a) normal control group: there were brown positive expressions of A*β*_1–42_ inside the cell but negative outside the cell; (b) model group: there were positive expressions of A*β*_1–42_ and plaque deposits with compactness outside the cell; (c) EA group: there were a few diffuse senile plaques.

In the normal control group, there were brown positive expressions of A*β*_1–42_ inside the cell and negative ones outside the cell. In the model group, there were positive expressions of A*β*_1–42_ and plaque deposits with compactness outside the cell. Compared with the model group, the expression of A*β*_1–42_ in EA group was significantly weakened, and there were a few diffuse senile plaques (see Figure 3).

#### 5.2.2. Laser Confocal Imaging of A*β*_1–42_ and LRP1 in Hippocampus

**Figure 4:**
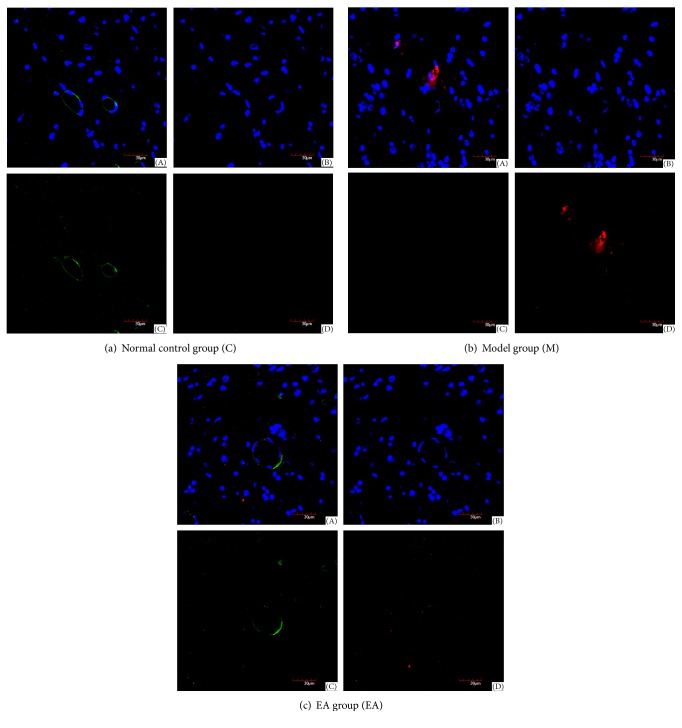
Laser confocal imaging of A*β*_1–42_ and LRP1 in hippocampus (original magnification ×600): (A) stack imaging of the three; (B) imaging of cell nucleus; (C) LRP1 labeled with green fluorescence; (D) A*β*_1–42_ labeled with red fluorescence; (a) in normal control group, plaque deposits of A*β*_1–42_ were not found, while LRP1 was expressed mostly around the vascular endothelial cells; (b) in model group, there were dense-core plaques deposited, while less LRP1 was expressed; (c) in EA group, the senile plaques were relatively reduced, and there were only some diffuse plaques, while the expression of LRP1 was more than that of model group.

In the normal control group, plaque deposits of A*β*_1–42_ were not found, while LRP1 was expressed mostly around the vascular endothelial cells. In the model group, there were dense-core plaques deposited, while less LRP1 was expressed. In EA group, the senile plaques were relatively reduced, and there were only some diffuse plaques, while the expression of LRP1 was more than that of model group. A*β*_1–42_, LRP1, and cell nucleus were labeled with laser confocal imaging (see Figure 4).

#### 5.2.3. Effect of EA on A*β*_1–40_ and A*β*_1–42_ Expression

The ELISA results were shown in Table 3. LSD test was used for pairwise comparison. Results showed that A*β*_1–42_ and A*β*_1–40_ expression levels and A*β*_1–42_/A*β*_1–40_ values in the model group were all higher than those of the normal control group (*P* < 0.01); A*β*_1–42_ and A*β*_1–40_ expression levels in the EA group were lower than those in the model group (*P* < 0.01), while there was no significant difference for A*β*_1–42_/A*β*_1–40_ between the EA and model groups.

#### 5.2.4. Effect of EA on Relative Expression Level of LRP1

**Figure 5:**
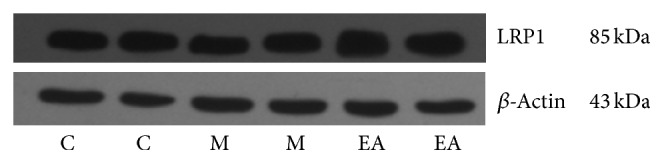
Effect of EA on relative expression level of LRP1.

Western blotting results of LRP1 were shown in Table 4 and Figure 5. The LRP1/*β*-actin gray value was the relative expression level of LRP1.

*LRP1/Actin*. There was a significant difference between the model group and the normal control group (*P* < 0.01), and the EA group was significantly higher than the model group (*P* < 0.01) (see Table 4).

Table 5 shows the ELISA test results of LRP1 expressions. There was a significant difference between the model group and the normal control group (*P* < 0.01). There was no significant difference between the EA group and the model group (*P* > 0.05), but the EA group was higher than the model group.

## 6. Discussions

The disordered clearance of A*β* is the main pathogenesis of AD. A*β* is cleared out through the BBB or degraded through the pathway of brain cell fluid drainage [31, 32]. The A*β* level between the brain parenchyma and brain interstitial fluid is inversely proportional to the age of mice. For the older mice, the A*β* level of brain parenchyma increases, while the A*β* level of brain interstitial fluid decreases [33]. The experimental study has found that LRP1 can transport A*β* out from BBB to be involved in the clearance of A*β* vessels [34], and the A*β* level can be decreased by transporting it.

In 1997, Narita et al. [35] discovered that there was a correlation between LRP1 and AD. However, studies on the pathogenesis of AD have shown that the functions of LRP1 are complicated, and some results are contradictory. On one hand, LRP1 can internalize APP and decompose it as A*β* in organelle lumen; on the other hand, LRP1 on neuronal membrane can also perform enzymatic degradation through the endocytosis of A*β* with its ligand *α*2M and ApoE; meanwhile, LRP1 on brain microvascular endothelial cells may mediate the outflow transport of A*β* across the BBB. Therefore, LRP1 is correlated to the generation and clearance of A*β* in some degree [36–38]. Studies have indicated that, for the AD model, the transportation of A*β* across the BBB mediated by LRP1 in the brain is damaged [39, 40]. The inhibition of LRP1 expression in brain of healthy mice could decrease the outflow rate of A*β* in brain by 30% [41] and prompt the sedimentation of A*β* in brain.

Studies have shown that, by injecting iodine-labeled A*β* into the brain, the rapid transport process of A*β* across the BBB can be observed [42]. This process can be inhibited by LRP1's sensitive inhibitor (RAP) and *α*2 macroglobulin. All these evidences prove that LRP1 on the BBB may be the main carrier of A*β* transported out from the brain; that is, LRP1 is involved in the clearance of A*β*, and it also has a negligible effect on the prevention of AD symptoms.

Experiments have shown that electroacupuncture can improve learning and memory abilities in transgenic mice and decrease the A*β* level in brain. The mechanism may be related to the effect of electricity on the cerebral microvessels and the A*β* transport receptor LRP1 [13, 18, 43].

Our results show that, in the water maze escape latency, mice in the normal control group and the EA group had separately retained the spatial memory in the 2nd and 3rd days, and the escape latency time shortened with the increase in days. It indicates that EA has improved the AD models' ability of spatial learning and memory. ELISA testing displayed that the levels of A*β* were significantly decreased, which shows a consistency with the results of immunohistochemistry and laser confocal imaging. This may be a mechanism of EA's improvement of the memory of AD mice.

The AD animal models are APPswe/PS1dE9 double transgenic mice prepared by transferring the human mutated genes APP and PS1 into mice to raise the A*β* levels in brain so as to cause a series of pathological lesions of AD. This model assumes that A*β* is the main pathological pathogenic factor. It was reported previously that the escape latency time in spatial probe test increased for 3-month-old APPswe/PS1dE9 double transgenic mice [44], while in the preliminary study of this experiment, it was found that the Morris water maze escape latency time in EA group and model group was not significantly different from that in normal control group for 5-month-old double transgenic mice. The water maze experiments of Li et al. [26] have indicated that the learning and memory disorders begin to appear at the age of 7-8 months.

This study has shown that EA can improve the spatial learning and memory of 7-month-old APP/PS1 transgenic mice in the model group which have been shown to have both learning and memory disorders.

Our research group has previously done a study of EA on GV 20 (*băi huì*) and KI 1 (*yŏng quán*) as the intervention to APPV717I AD mice. EA could intervene in the behavior of 11-month-old APP single transgenic AD mice, neuronal changes, and A*β* protein expression in brain, especially where there is a decreasing trend of A*β* sedimentation in the hippocampus microvessels [43]. EA therapy can regulate the APP/PS1 double transgenic mice hippocampus ultrastructure [45]. APP/PS1 double transgenic mice were used in this study which could cause earlier pathological manifestations such as A*β* sedimentation, senile plaques, neuronal loss, and behavioral obstacles of cognitive performance compared to single-transgenic mice, so that the length of the EA experiment cycle can be shortened. The appearance of senile plaques on brain cortex for 7-month-old APP/PS1 transgenic mice has indicated an early intervention of EA.

The focus of this study is on the effect of EA on LRP1, which is the key receptor in A*β* clearance of vessels in brains of the models. LRP1 mainly exists in cerebral vascular endothelial cells, vascular smooth muscle cells, and other cells including neurons, astrocytes, and smooth muscle cells [46]. In this study, the laser scanning confocal microscope was applied to observe the coexpression of LRP1 and A*β* in the hippocampus, proving that LRP1 is expressed on the microvascular endothelial cells.

This experiment showed that the A*β* levels in the model group increased, while EA can decrease A*β* levels. Furthermore, LRP1 levels in the model group decreased, while EA increased LRP1 levels. Therefore, the mechanism of EA's involvement in improving learning and memory may be its ability to increase LRP1 levels, thus increasing the clearance of A*β*.

It has been reported that A*β* can be directly transported by LRP1, and studies have also shown that A*β* can be only transported after forming complexes with other ligands of LRP1 such as ApoE [47]. Does EA regulate another mechanism of ApoE and so forth by increasing the rate and quantity of combination of A*β* and LRP1 and further decrease A*β*? Further studies of possible mechanisms are required.

In summary, this study has indicated that EA can improve learning and memory of mice. A*β* levels were shown to be significantly lower in the EA group compared to the model group, while LRP1 levels were also significantly higher in the EA group, which may indicate that the decreasing of A*β* is related to the transportation of LRP1; therefore EA may be involved in regulating LRP1 functions. Enhancing the transportation of A*β* protein and decreasing the A*β* levels may be a functional way to treat dementia using EA, but still further evidence is needed.

## 7. Conclusions

EA therapy can improve both learning and memory capabilities in APP/PS1 transgenic mice. The underlying mechanism may be due to the upregulation of the A*β* transport receptor LRP1, thus acting on learning and memory by contributing to decreased levels of A*β* in the hippocampus.

## Figures and Tables

**Table 1:** Comparison of escape latency time in each group in Morris water maze place navigation test ($\overset{-}{x}\pm s$, s, *n* = 20).

Groups	Cases	Day 1	Day 2	Day 3	Day 4
Normal control group (C)	20	54.19 ± 6.13	45.96 ± 11.64^▲▲^	40.33 ± 14.89^▲▲^	37.93 ± 13.78^▲▲^
Model group (M)	20	58.32 ± 4.6^*※※*^	60.00 ± 0.00^*※※*^	55.39 ± 8.30^*※※*^	54.65 ± 9.87^*※※*^
EA group (EA)	20	58.14 ± 5.07	57.05 ± 5.72^*∗∗*^	52.45 ± 9.40^▲▲^	48.65 ± 12.71^*∗*▲▲^

Notes: ^*※※*^compared with the normal control group, *P* < 0.01; ^*∗*^compared with the model group, *P* < 0.05; ^*∗∗*^compared with the model group, *P* < 0.01; ^▲▲^compared with the first day of the same group, *P* < 0.01.

**Table 2:** Comparison of platform crossover number and swimming distance in platform quadrant of each group in Morris water maze spatial probe test ($\overset{-}{x}\pm s$, time, cm, *n* = 20).

Groups	Cases	Platform crossover number	Swimming distance in platform quadrant
Normal control group (C)	20	1.74 ± 1.4	367.35 ± 142.89
Model group (M)	20	0.50 ± 0.44^*※※*^	192.46 ± 72.00^*※※*^
EA group (EA)	20	0.90 ± 0.71^*∗*^	296.61 ± 105.84^*∗∗*^

Notes: ^*※※*^compared with the normal control group, *P* < 0.01; ^*∗*^compared with the model group, *P* < 0.05; ^*∗∗*^compared with the model group, *P* < 0.05.

**Table 3:** Effect of EA on Aβ_1–42_ and Aβ_1–40_ expression ($\overset{-}{x}\pm s$, pg/mg, *n* = 6).

Groups	Cases	Aβ_1–42_	Aβ_1–40_	Aβ_1–42_/Aβ_1–40_
Normal control group (C)	6	0.14 ± 0.38	0.077 ± 0.01	1.79 ± 0.61
Model group (M)	6	6119.76 ± 670.13^*※※*^	801.05 ± 219.24^*※※*^	7.97 ± 1.61^*※※*^
EA group (EA)	6	1326.58 ± 501.40^*∗∗*^	297.05 ± 112.89^*∗∗*^	7.02 ± 1.73

Notes: ^*※※*^compared with the normal control group, *P* < 0.01; ^*∗∗*^compared with the model group, *P* < 0.01.

**Table 4:** Effect of EA on relative expression level of LRP1 ($\overset{-}{x}\pm s$, *n* = 6).

Groups	Cases	LRP1 gray value (×10^4^)	Actin gray value (×10^4^)	LRP1/Actin
Normal control group (C)	6	6.77 ± 1.17	11.16 ± 0.86	0.60 ± 0.08
Model group (M)	6	3.87 ± 0.76^*※※*^	11.77 ± 1.24	0.33 ± 0.07^*※※*^
EA group (EA)	6	6.34 ± 1.32^*∗∗*^	11.86 ± 0.74	0.53 ± 0.10^*∗∗*^

Notes: ^*※※*^compared with the normal control group, *P* < 0.01; ^*∗∗*^compared with the model group, *P* < 0.01.

**Table 5:** Effect of EA on LRP1 expression ($\overset{-}{x}\pm s$, pg/mg, *n* = 9).

Groups	Cases	LRP1
Normal control group (C)	9	0.21 ± 0.25
Model group (M)	9	0.17 ± 0.1^*※※*^
EA group (EA)	9	0.19 ± 0.11

Notes: ^*※※*^compared with the normal control group, *P* < 0.01.
